# Cerebral Protection With Deep Hypothermic Circulatory Arrest During Total Arch Replacement Using the Arch-First Technique for Acute Aortic Dissection

**DOI:** 10.7759/cureus.66640

**Published:** 2024-08-11

**Authors:** Kimiaki Okada, Sohsyu Kotani, Keisuke Ozawa, Goro Kishinami, Akiyoshi Yamamoto, Yasunori Cho

**Affiliations:** 1 Cardiovascular Surgery, Tokai University School of Medicine, Kanagawa, JPN

**Keywords:** dissection with patent false lumen, stroke due to air embolism, acute type a aortic dissection, deep hypothermic circulatory arrest(dhca), total arch replacement

## Abstract

Objectives: Stroke remains a serious complication after total arch replacement (TAR). To prevent this, deep hypothermia is commonly employed during TAR. We evaluated the effectiveness of cerebral protection using deep hypothermic circulatory arrest (DHCA) during TAR with the arch-first technique, focusing particularly on patients with acute aortic dissection (AAD).

Methods: This retrospective study included 109 consecutive patients with AAD who underwent emergency TAR using the arch-first technique under DHCA, and 147 patients with non-ruptured aneurysm who underwent scheduled TAR using the same technique between October 2009 and July 2022. We reviewed these patients for major adverse events, including stroke and 30-day mortality after surgery. We also analyzed the impact of clinical variables and anatomical features on the occurrence of newly developed stroke after TAR in patients with AAD.

Results: A newly developed stroke after TAR occurred in 11 (10.1%) patients with AAD. These were attributed to embolism in eight patients, malperfusion in two patients (including one who had been comatose), and low output syndrome in one patient. A stroke occurred in 3 (2.0%) patients with aneurysm, all due to embolism (P = 0.005). The DHCA time was 37 ± 7 minutes for patients with AAD and 36 ± 6 minutes for patients with aneurysm (P = 0.122). The 30-day mortality rate was 10 (9.2%) for patients with AAD and 2 (1.4%) for patients with aneurysm (P = 0.003). In our multivariable analysis, arch vessel dissection with a patent false lumen (double-barreled dissection) was the only significant predictor of newly developed stroke after TAR for AAD (odds ratio, 33.02; P < 0.001).

Conclusions: Patients with aneurysm undergoing TAR using the arch-first technique under DHCA experienced significantly better outcomes, in terms of newly developed stroke and 30-day mortality, than those with AAD. Cerebral protection with DHCA during TAR using the arch-first technique continues to be a viable option. Newly developed stroke in patients undergoing TAR for AAD appears to be associated with air emboli deriving from the residual dissection with a patent false lumen in the repaired arch vessels.

## Introduction

Clinical outcomes after total arch replacement (TAR) have improved in recent decades [1,2], largely due to cerebral protection methods such as deep hypothermia associated with retrograde or antegrade cerebral perfusion, as well as recent advancements in aortic surgery [3-10]. Unfortunately, stroke after TAR remains a serious complication and is a leading cause of mortality and morbidity, particularly in patients with acute aortic dissection (AAD), who experience a higher incidence of stroke following surgery [1,2,11]. Several distinct features are related to cerebral malperfusion in AAD and reperfusion injury following TAR. However, surgical stress from cardiopulmonary bypass and prolonged hypothermic circulatory arrest times places patients at significant risk of stroke [12,13]. To address unexpected organ malperfusion concomitant with AAD, central cannulation in the ascending aorta has been established under controlled blood pressure with superior and inferior vena cava drainage. To prevent newly developed stroke, we have utilized deep hypothermic circulatory arrest (DHCA) during the arch-first technique, in which arch vessels are reconstructed using a branched arch graft, providing an effective surgical view prior to distal arch anastomosis in TAR [3,4,14]. Retrograde cerebral perfusion is initiated at the end of arch vessel anastomoses to evacuate emboli such as air, thrombi, and debris [15]. The cause of stroke associated with DHCA during TAR in patients with AAD, however, remains unclear. In the present study, we retrospectively reviewed patients with AAD and nonruptured aneurysm who underwent TAR using the arch-first technique during DHCA. This review focused on newly developed stroke and 30-day mortality. Specifically, we analyzed the effects of clinical variables and anatomical features on newly developed stroke following TAR in patients with AAD.

This article was previously presented as a meeting abstract at the AATS Aortic Symposium 2024 on April 25 and 26, 2024.

## Materials and methods

Study design and patients

From October 2009 to July 2022, 109 consecutive patients with AAD underwent TAR using the arch-first technique under DHCA on an emergency basis, and 147 consecutive patients with nonruptured arch aneurysm underwent scheduled TAR, also using the same technique. These patients comprise the study population and were retrospectively reviewed for outcomes following TAR with DHCA. We also examined demographic characteristics, perioperative clinical variables, and imaging studies for anatomical features. The primary endpoint was newly developed stroke, assessed by computed tomography (CT) or magnetic resonance imaging (MRI). The secondary endpoint included 30-day mortality or operative mortality, with operative mortality defined as 30-day mortality and/or in-hospital mortality.

The Institutional Review Board of Tokai University Hospital approved the study protocol and the publication of data (22R-027). Informed written consent for the publication of study data was obtained from all patients.

Neurologic evaluation 

**Figure 1:**
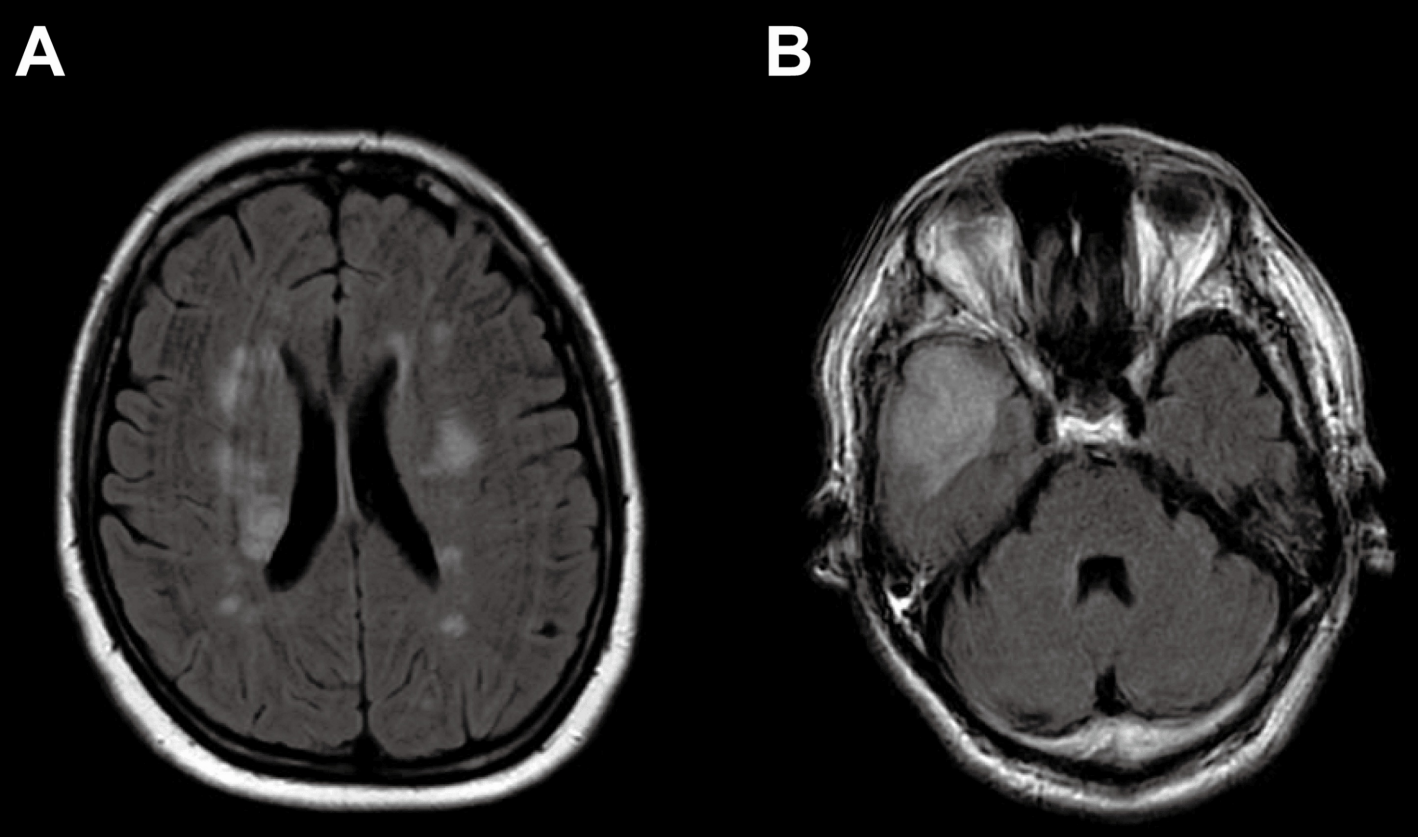
Postoperative MRI of the head in patients with newly developed stroke after TAR using DHCA. (A) Well-margined lacunar infarctions in multiple vascular territories, which were more prevalent in embolic stroke. (B) Cerebral malperfusion, exclusively defined as infarction in the territory of the cerebral artery associated with AAD in the presence of high-grade stenosis or lack of opacification through the arch vessels. MRI, magnetic resonance imaging; TAR, total arch replacement; DHCA, deep hypothermic circulatory arrest; AAD, acute aortic dissection.

All patients underwent preoperative head CT and/or MRI, either to create a reference image or due to the presence of neurologic symptoms in patients with AAD. Neurologic evaluations were performed by a neurologist or emergency physician. Coma and paralysis were classified as neurologic deficits. The level of impaired consciousness was assessed using the Glasgow Coma Scale (GCS); a GCS score of 8 or less was defined as a comatose state. Motor function in the affected areas was evaluated using the manual muscle test (MMT). Patients with AAD who exhibited transient neurologic symptoms or neurologic deficits without radiographically evident findings were still considered for immediate aortic repair. Newly developed stroke was defined as the onset of a neurologic deficit lasting longer than 72 hours, accompanied by new pathological findings on CT or MRI. Radiographically, well-margined lacunar infarctions in multiple vascular territories were more common in cases of embolic stroke (Figure 1A). Cerebral malperfusion was specifically defined as infarction within the territory of the cerebral artery associated with AAD, in the presence of high-grade stenosis or lack of opacification through the arch vessels (Figure 1B), sometimes presenting as watershed infarction [16].

Surgical technique

Our standard operation involves sternotomy with cardiopulmonary bypass and DHCA, as previously described [12]. In patients with AAD, cardiopulmonary bypass was established using echo-guided central cannulation in the ascending aorta, following venous drainage from the superior and inferior vena cava. Regional oxygen saturation in the bilateral frontal regions was monitored using near-infrared spectroscopy to ensure adequate cerebral blood flow. Patients were placed in the Trendelenburg position, and circulatory arrest was initiated when the drained blood temperature reached 17.5°C. TAR was performed using the arch-first technique, where the arch vessels are repaired first with a Dacron 4-branched graft (Hemashield Platinum Plus Woven Double Velour, 4 branch, Intervascular SAS, La Ciotat, France), to minimize the duration of circulatory arrest [14]. Briefly, the aortic arch was opened, and the arch vessels were anastomosed in the following order: the left subclavian artery, the left common carotid artery, and the brachiocephalic artery, prior to the distal anastomosis. After the arch vessel anastomosis was completed, retrograde cerebral perfusion was performed via the superior vena cava with hypothermia at 20°C to flush out air, thrombi, and atheromatous debris. Cerebral perfusion was then established through the fourth graft branch, with clamps applied to both ends of the graft. The distal anastomosis was performed by open distal anastomosis using the elephant trunk technique while lower body circulation was arrested. Myocardial protection was achieved through antegrade delivery of cold blood cardioplegia, followed by proximal anastomosis. In patients with AAD, the aortic valve was resuspended. No patients underwent concomitant procedures such as aortic root repair or coronary artery bypass grafting.

Statistical analysis

Continuous variables, confirmed as normally distributed by the F-test, are presented as mean ± standard deviation or as median with interquartile range, as appropriate. Patient demographics, surgical demographics, and outcomes after TAR using DHCA were compared between patients with AAD and those with aneurysm using the unpaired t-test. Categorical data are expressed as percentages, and proportions were analyzed using the chi-square test or Fisher’s exact test, as appropriate. Univariable and multivariable analyses were performed using the logistic regression model to assess the relationships between anatomical features of AAD and newly developed stroke after TAR using DHCA, adjusting for confounding variables such as patient and surgical demographics. Differences were considered significant at P < 0.05. All statistical analyses were performed using IBM SPSS Statistics for Windows, Version 24 (Released 2016; IBM Corp., Armonk, New York).

## Results

Patient demographics 

Table 1 sets out the preoperative demographics of patients undergoing TAR using the arch-first technique under DHCA. Patients with AAD were significantly younger (63 ± 11 vs 74 ± 9 years, P < 0.001) and had a lower comorbidity of chronic obstructive pulmonary disease (0.9% vs 9.5%, P = 0.004) compared to those with aneurysm. There was no significant difference in other comorbidities, such as hypertension (97.2% vs 98.6%, P = 0.426), diabetes mellitus (18.3% vs 24.5%, P = 0.240), and hemodialysis (0.9% vs 2.0%, P = 0.474). All patients with AAD showed dissection in the ascending aorta. Of these, 52 (47.7%) patients had a patent false lumen, and the other 57 (52.3%) had thrombi in the false lumen, including 24 (22.0%) patients with thrombosed dissection. The arch vessels were dissected in 61 (56.0%) patients. Of these, 21 (19.3%) had a patent false lumen, and 47 (43.1%) had a thrombosed false lumen. Twelve (11.0%) patients presented in a comatose state. Patients with AAD had a significantly lower left ventricular ejection fraction than those with aneurysm (65.4 ± 10.5% vs 68.9 ± 9.0%, P = 0.004).

**Table 1:** Preoperative demographics of patients who underwent TAR using arch-first technique under DHCA Categorical data are presented as numbers (%), and continuous data are presented as mean ± standard deviation. *P value calculated using the chi-square test or Fisher’s exact test for categorical variables, as appropriate, and the unpaired t-test for continuous variables. TAR, total arch replacement; DHCA, deep hypothermic circulatory arrest; BSA, body surface area; COPD, chronic obstructive pulmonary disease.

Demographics	Acute aortic dissection (n = 109)	Aneurysm (n = 147)	P-value*
Age	63 ± 11	74 ± 9	<0.001
Gender, male	59 (54.1%)	121 (82.3%)	<0.001
BSA (m^2^)	1.68 ± 0.21	1.65 ± 0.16	0.243
Hypertension	106 (97.2%)	145 (98.6%)	0.426
Diabetes mellitus	20 (18.3%)	36 (24.5%)	0.240
Hemodialysis	1 (0.9%)	3 (2.0%)	0.474
COPD	1 (0.9%)	14 (9.5%)	0.004
Current smoker	13 (11.9%)	9 (6.1%)	0.010
Serum creatinine (mg/dL)	0.95 ± 0.54	1.19 ± 0.88	0.012
Ejection fraction (%)	65.4 ± 10.5	68.9 ± 9.0	0.004
Comatose state	12 (11.0%)	0	<0.001
Dissected ascending aorta	109 (100%)		
Patent false lumen	52 (47.7%)		
Thrombi in the false lumen	57 (52.3%)		
Dissected arch vessels	61 (56.0%)		
Patent false lumen	21 (19.3%)		
Thrombosed false lumen	47 (43.1%)		

Surgical demographics and outcomes

Table 2 sets out the surgical demographics and outcomes of patients undergoing TAR using the arch-first technique under DHCA. Cardiopulmonary bypass was established via the ascending aorta or aortic arch in all patients undergoing TAR using DHCA, including those with AAD, for whom cardiopulmonary bypass was established with echo-guided central cannulation in the ascending aorta. No patients with AAD showed cerebral desaturation after the establishment of cardiopulmonary bypass, and we therefore did not perform true lumen reperfusion via the right subclavian arterial cannulation. Seven (6.7%) patients with AAD required femoral cannulation due to lower limb ischemia in five patients and suspected mesenteric ischemia in two patients. Upon completion of the anastomosis of the arch vessels, retrograde cerebral perfusion for backflushing was also performed in all patients. There was no significant difference in circulatory arrest time between patients with AAD and those with aneurysm (37 ± 8 vs 36 ± 6 minutes, P = 0.122). Cardiopulmonary bypass time was significantly longer in patients with AAD than in those with aneurysm (269 ± 76 vs 210 ± 34 minutes, P < 0.001), associated with a higher frequency of low output syndrome (11.9% vs 0.7%, P < 0.001). A newly developed stroke after TAR occurred in 11 (10.1%) patients with AAD. These were due to embolism in eight patients, malperfusion in two patients (including one who had been in a comatose state), and low output syndrome (LOS) in one patient. A newly developed stroke occurred in three (2.0%) patients with aneurysm (at a lower frequency, P = 0.005), all three due to embolism. Thirty-day mortality occurred in 10 (9.2%) patients with AAD, due to LOS in three patients, stroke in two, pneumonia in two, colon ischemia in one, disseminated intravascular coagulation in one, and rupture of the proximal anastomosis in one. Thirty-day mortality occurred in 2 (1.4%) patients with aneurysm, due to pneumonia and colon ischemia (at lower frequency, P = 0.003).

**Table 2:** Surgical demographics and outcomes of patients who underwent TAR using arch-first technique under DHCA Categorical data are presented as numbers (%), and continuous data are presented as mean ± standard deviation. *P-value calculated using the chi-square test or Fisher’s exact test for categorical variables, as appropriate, and the unpaired t-test for continuous variables. ^†^Echocardiography-guided central cannulation in the ascending aorta was performed for patients with acute aortic dissection. TAR, total arch replacement; DHCA, deep hypothermic circulatory arrest.

Demographics	Acute aortic dissection (n = 109)	Aneurysm (n = 147)	P value*
Arterial cannulation site			
Ascending†	109 (100%)	147 (100%)	-
Right subclavian	0	0	-
Femoral	7 (6.4%)	0	<0.001
Adjunct cerebral protection			
Antegrade	0	0	-
Retrograde for backflushing	109 (100%)	147 (100%)	-
Circulatory arrest time (min)	37 ± 8	36 ± 6	0.122
Cardiopulmonary bypass time (min)	269 ± 76	210 ± 34	<0.001
Low output syndrome	13 (11.9%)	1 (0.7%)	<0.001
Newly developed stroke	11 (10.1%)	3 (2.0%)	0.005
Embolism	8 (7.3%)	3 (2.0%)	
Malperfusion	2 (1.8%)	0	
Low output syndrome	1 (0.9%)	0	
30-day mortality	10 (9.2%)	2 (1.4%)	0.003
Operative mortality	13 (11.9%)	4 (2.7%)	0.003

Stroke after TAR using DHCA for patients with AAD

Table 3 summarizes our patients with newly developed stroke after TAR using the arch-first technique under DHCA for AAD. Seven of the eight patients with stroke due to embolism had a dissection with a patent false lumen in the arch vessels. Of these, five patients did not exhibit thrombus in the ascending aorta or in the dissected arch vessels. The median GCS of all 11 patients with stroke was 12 (range 7-15), and the median MMT was 2 (range 0-4). Ten of the 11 patients were transferred to rehabilitation hospitals. One patient with a thrombosed false lumen in the arch vessels, who had not shown neurologic symptoms or abnormal CT findings preoperatively, developed severe cerebral edema due to malperfusion during TAR and died in the hospital. In this patient, neither bilateral nor ipsilateral oxygen desaturation in the frontal regions was observed perioperatively.

**Table 3:** Patients with newly developed stroke after TAR using arch-first technique under DHCA for AAD *Patient died of cerebral edema. TAR, total arch replacement; DHCA, deep hypothermic circulatory arrest; AAD, acute aortic dissection; Preop, preoperative; Asc Ao, ascending aorta; CA, circulation arrest time; Periop, perioperative; LOS, low output syndrome; GCS, Glasgow Coma Scale; MMT, manual muscle test; Double-barreled, dissection with patent false lumen; Thrombosed, dissection with thrombosed false lumen.

Age/gender	Cause of stroke	Laterality of stroke	Preop. coma	Thrombi Asc Ao.	Dissection in the arch vessels	CA (min)	Periop. LOS	GCS	MMT	Outcomes
57, M	Embolism	Bilateral	-	-	Double-barreled	48	-	14	2	Transfer
60, M	Embolism	Bilateral	-	-	Double-barreled	43	-	14	4	Transfer
79, F	Embolism	Bilateral	-	-	Double-barreled	31	-	14	4	Transfer
53, M	Embolism	Bilateral	-	-	Double-barreled	44	-	14	4	Transfer
66, F	Embolism	Right	-	-	Double-barreled	23	-	15	4	Transfer
48, F	Embolism	Bilateral	-	+	Double-barreled	46	-	3	0	Transfer
68, M	Embolism	Bilateral	-	+	Double-barreled	28	-	11	2	Transfer
61, F	Embolism	Bilateral	-	+	None	35	-	2	0	Transfer
78, F	Malperfusion	Right	+	-	Thrombosed	28	-	12	2	Transfer
68, F	Malperfusion	Bilateral	-	-	Thrombosed	29	-	3	0	Dead*
55, M	LOS	Left	-	-	Double-barreled	48	+	10	4	Transfer

In the logistic regression model, dissection with a patent false lumen in the arch vessels was significantly associated with newly developed stroke after TAR using the arch-first technique under DHCA for patients with AAD in both univariable (odds ratio (OR), 22.75; 95% confidence interval (CI), 5.34-96.92; P < 0.001) and multivariable analysis (OR, 33.02; 95% CI, 4.33-252.1; P < 0.001); see Table 4.

**Table 4:** Logistic regression analysis of newly developed stroke after TAR using arch-first technique under DHCA for AAD TAR, total arch replacement; AAD, acute aortic dissection; OR, odds ratio; CI, confidence interval; DHCA, deep hypothermic circulatory arrest.

Demographics	Univariable		Multivariable	
	OR (95% CI)	P-value	OR (95% CI)	P-value
Age	1.00 (0.94–1.05)	0.867	0.97 (0.90–1.06)	0.517
Gender, male	0.68 (0.19–2.38)	0.544	0.57 (0.12–2.78)	0.483
Comatose state	0.79 (0.09–6.79)	0.831	0.39 (0.03–5.56)	0.487
Thrombi in the ascending aorta	0.60 (0.16–2.16)	0.431	1.51 (0.27–8.55)	0.641
Dissection in the arch vessels				
Patent false lumen	22.75 (5.34–96.92)	<0.001	33.02 (4.33–252.1)	<0.001
Thrombosed false lumen	0.26 (0.05–1.27)	0.097	1.10 (0.14–8.93)	0.926
Circulatory arrest time	1.02 (0.95–1.11)	0.558	0.98 (0.88–1.09)	0.704
Low output syndrome	0.72 (0.08–6.11)	0.761	1.74 (0.13–24.08)	0.680

## Discussion

The main findings of this retrospective study are as follows. (1) Patients with aneurysm undergoing TAR using the arch-first technique under DHCA experienced significantly better outcomes in terms of newly developed stroke (2.0% vs 10.1%, P = 0.005) and 30-day mortality (1.4% vs 9.2%, P = 0.003) than patients with AAD. (2) Newly developed stroke after TAR occurred in 11 (10.1%) patients with AAD, primarily due to embolism in eight patients. In our multivariable analysis, dissection with a patent false lumen in the arch vessels (OR 33.02, CI (4.33-252.1), P < 0.001) was the only significant predictor of newly developed stroke after TAR for AAD.

Newly developed stroke after TAR remains a serious complication and is a leading cause of mortality and morbidity [1,2,9,11]. This is strongly associated with TAR for AAD on an emergent basis [17,18]. Our data show that patients with AAD undergoing TAR using the arch-first technique under DHCA had a significantly higher rate of newly developed stroke (10.1% vs 2.0%, P = 0.005) and operative mortality (11.9% vs 2.7%, P = 0.003) than patients with aneurysm. These rates are, however, comparable to newly developed stroke rates from the Japan Cardiovascular Surgery Database: 11.9% in patients with AAD undergoing TAR and 6.3% in those with aneurysm, with operative mortality rates of 11.2% for AAD and 5.1% for aneurysm [1]. We therefore believe that cerebral protection with DHCA during TAR using the arch-first technique continues to be a viable option.

In the present study, we consider that the newly developed stroke rate of 10.1% after TAR using the arch-first technique under DHCA was quite high in patients with AAD. The causes of stroke were embolism, such as air and thrombus, in eight patients, malperfusion in two patients, and LOS in one patient. Stroke after AAD repair is associated with higher operative mortality, higher postoperative renal and respiratory failure, longer intensive care unit stays, longer hospital stays, and reduced long-term survival [19,20]. In particular, patients with cerebral malperfusion resulting from arch vessel involvement in AAD have been reported to have a poor prognosis in relation to newly developed stroke and mortality [21,22].

Two of our patients had newly developed stroke associated with cerebral malperfusion resulting in infarction in the territory of the cerebral artery associated with AAD, and both exhibited thrombosed dissection in the common carotid arteries (Table 3). Internal carotid artery obstruction in the presence of arch vessel dissection may be a surrogate marker for poor neurologic outcomes regardless of the surgical approach, whereas common carotid artery obstruction or comatose state should not rule out the surgical procedure. We therefore promptly established cardiopulmonary bypass using echo-guided central cannulation in the ascending aorta to prevent newly developed stroke in patients with AAD. We believe that this has potential as a rapid and reliable perfusion route during surgery, even in patients who were in a comatose state without any CT findings of brain injury. Recently, brain computed tomography was reported to detect irreversible ischemic core [23]. This could be helpful in guiding critical decisions in preoperative patients with AAD who show neurologic symptoms.

Svensson and colleagues [4] reported that the occurrence of stroke increased after 40 minutes of DHCA, and the mortality rate increased markedly after 65 minutes of DHCA. In the present study, we observed five patients with AAD developing new strokes whose DHCA duration was longer than 40 minutes (Table 3), although they did not show cerebral infarction in the watershed region, which is typically associated with prolonged DHCA. Moreover, there was no statistical difference in DHCA time between patients with AAD and those with aneurysm (37 ± 8 vs 36 ± 6 minutes, P = 0.122) (Table 2). We also observed one patient with newly developed stroke among 13 (11.9%) patients who experienced LOS after aortic repair for AAD. The cause of all LOS cases was prolonged cardiac arrest time required to establish adequate hemostasis at the distal anastomosis. In the multivariable analysis, circulatory arrest time (OR, 0.98; P = 0.704) and LOS (OR, 1.74; P = 0.680) were not significant predictors of newly developed stroke after TAR for AAD, although dissection with a patent false lumen in the arch vessels (OR, 33.02; P < 0.001) was found to be the only significant predictor (Table 4).

**Figure 2:**
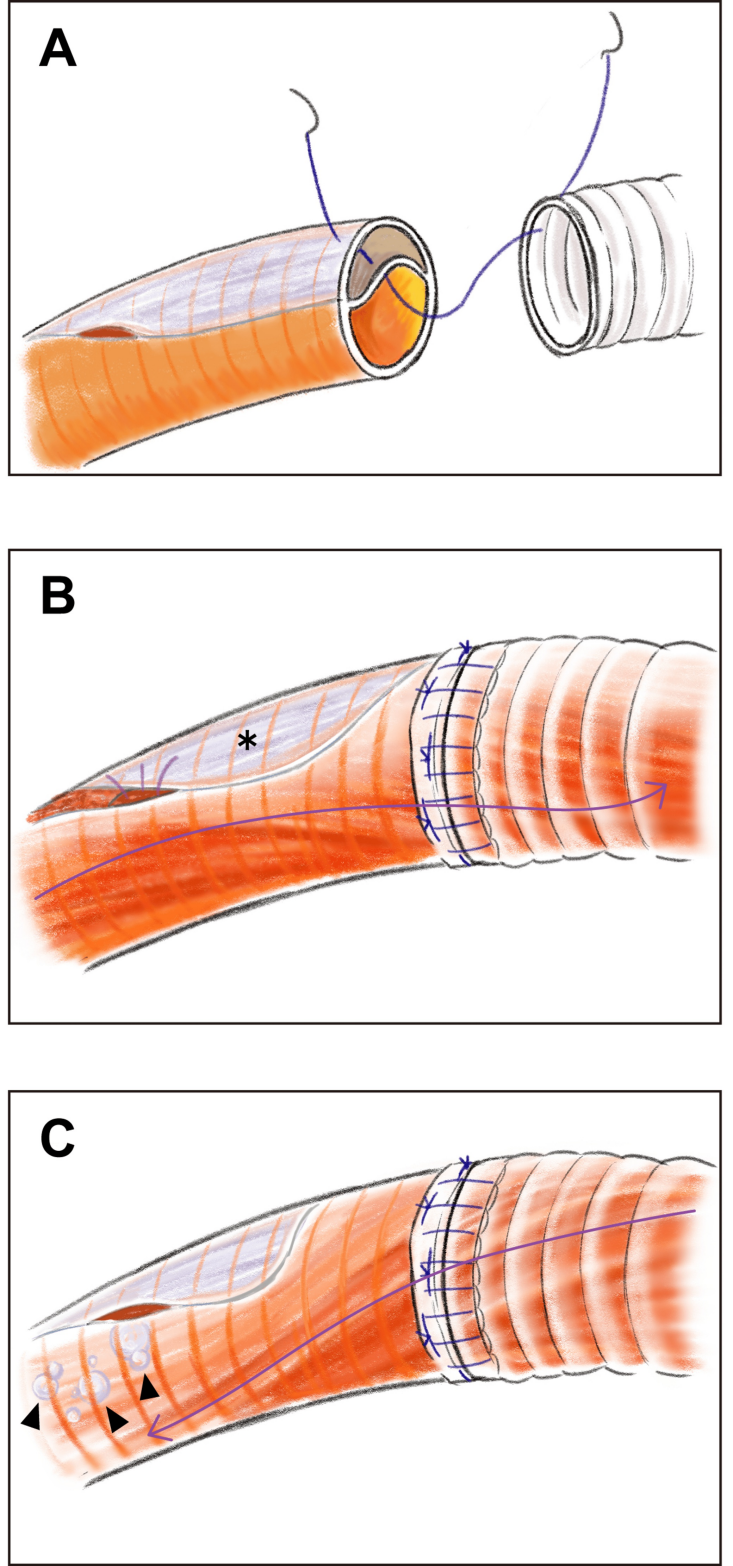
Dissected arch vessel anastomosis using the arch-first technique under DHCA (A) Dissected arch vessel anastomosis was performed using the arch-first technique under DHCA to secure a dry, quiet, and motionless surgical field. (B) Upon completion of the arch vessel anastomosis, retrograde cerebral perfusion was performed to flush out air, thrombi, and atheromatous debris. However, the expulsion of air (*) in the blind end of the patent false lumen after arch repair may be inadequate when using the same method of retrograde cerebral perfusion. (C) Antegrade cerebral perfusion was established via the graft branch. Air emboli (arrowheads) trapped in the cavity distal to the anastomosis were forced out through the re-entry into the brain. DHCA, deep hypothermic circulatory arrest. Original illustration by Kimiaki Okada.

We observed seven patients with dissection with a patent false lumen in the arch vessels who developed new strokes due to emboli (Table 3). We suggest that these were air emboli because, in five of the seven patients, we did not observe thrombi or atheromatous plaque in the ascending aorta or in the arch vessels. We have been using the arch-first technique under DHCA to secure a dry, quiet, and motionless surgical field unburdened by clamps and cannulas. Retrograde perfusion has been reported to be effective in the management of massive air embolism during cardiopulmonary bypass [14]. Consequently, when anastomosis of the arch vessels was completed, retrograde cerebral perfusion via the superior vena cava was performed with hypothermia in order to flush out not only air but also thrombi and atheromatous debris. Expulsion of air from the repaired arch vessels with a patent false lumen may, however, be inadequate in backflushing air via the same method of retrograde cerebral perfusion. Once antegrade cerebral perfusion was established via the graft branch, air emboli trapped in the cavity distal to the anastomosis could be forced out through the re-entry to the brain (Figure 2). Aortic arch vessel involvement in patients with AAD is reportedly associated with reduced cerebral blood flow and leads to a higher incidence of cerebral infarction [24]. We believe that this article is the first to suggest that trapped air emboli after repair of arch vessel dissection with a patent false lumen are associated with newly developed stroke in patients with AAD undergoing TAR using the arch-first technique under DHCA in the absence of continuous retrograde or antegrade cerebral perfusion.

Okita and colleagues [25] reported that DHCA, with or without continuous retrograde cerebral perfusion and antegrade cerebral perfusion, provides comparable clinical outcomes with regard to mortality and newly developed stroke rates, but DHCA with or without continuous retrograde cerebral perfusion leads to longer time in intensive care. Antegrade cerebral perfusion might be preferred as a method to protect the brain during complicated aortic arch procedures. Hypothermic circulatory arrest with continuous retrograde perfusion has been reported as a simple and useful adjunct for TAR, up to 80 minutes [26]. In the present study, TAR using the arch-first technique under DHCA gave satisfactory outcomes for patients with aneurysm. However, in patients with AAD and arch vessel dissection with a patent false lumen, other adjuncts for cerebral protection, such as antegrade and continuous retrograde cerebral perfusion, are recommended to prevent air embolism suggested in the arch vessel dissection with a patent false lumen.

Limitations

The present study is a single-center observational study with a limited study population. We did not confirm the presence of air emboli in the carotid artery during TAR. Regional oxygen saturation in the bilateral frontal regions was routinely monitored using near-infrared spectroscopy to confirm adequate cerebral blood flow. While we have detected desaturated oxygen levels when cerebral malperfusion occurs, it might be difficult to detect desaturation associated with lacunar infarctions in multiple vascular territories. We also utilized echocardiography to detect emboli in the carotid artery, although this method is not capable of detecting emboli in the cranial arteries. It would therefore be useful to evaluate other modalities for detecting emboli in the carotid artery. It is notable that we had 13 (11.9%) patients who experienced LOS after aortic repair for AAD. However, there is no uniform definition for LOS in current research and among clinicians [27]. In the present study, we defined LOS as the condition requiring prolonged mechanical support with cardiopulmonary bypass to maintain circulatory dynamics after TAR. Another study reported that 23% of patients experienced postoperative LOS (with no definition) after aortic repair for AAD [28]. Finally, we did not examine any patient group with AAD undergoing TAR using DHCA with antegrade or continuous retrograde cerebral perfusion. These results should be verified in large prospective randomized studies.

## Conclusions

Cerebral protection with DHCA during TAR continues to be an option, particularly for patients with aneurysm. Newly developed stroke in patients undergoing TAR for AAD appears to be associated with air emboli deriving from the residual dissection with a patent false lumen in the repaired arch vessels. In such patients, we therefore recommend other adjuncts for cerebral protection, such as antegrade and continuous retrograde cerebral perfusion, to avoid newly developed stroke.

## References

[1] Shimizu H, Hirahara N, Motomura N, Miyata H, Takamoto S (2019). Current status of cardiovascular surgery in Japan, 2015 and 2016: analysis of data from Japan Cardiovascular Surgery Database. 4-Thoracic aortic surgery. Gen Thorac Cardiovasc Surg.

[2] Okita Y, Kumamaru H, Motomura N, Miyata H, Takamoto S (2022). Current status of open surgery for acute type A aortic dissection in Japan. J Thorac Cardiovasc Surg.

[3] Griepp RB, Stinson EB, Hollingsworth JF, Buehler D (1975). Prosthetic replacement of the aortic arch. J Thorac Cardiovasc Surg.

[4] Svensson LG, Crawford ES, Hess KR, Coselli JS, Raskin S, Shenaq SA, Safi HJ (1993). Deep hypothermia with circulatory arrest. Determinants of stroke and early mortality in 656 patients. J Thorac Cardiovasc Surg.

[5] Ueda Y, Miki S, Kusuhara K, Okita Y, Tahata T, Yamanaka K (1990). Surgical treatment of aneurysm or dissection involving the ascending aorta and aortic arch, utilizing circulatory arrest and retrograde cerebral perfusion. J Cardiovasc Surg (Torino).

[6] Safi HJ, Letsou GV, Iliopoulos DC (1997). Impact of retrograde cerebral perfusion on ascending aortic and arch aneurysm repair. Ann Thorac Surg.

[7] Kazui T, Washiyama N, Muhammad BA, Terada H, Yamashita K, Takinami M, Tamiya Y (2000). Total arch replacement using aortic arch branched grafts with the aid of antegrade selective cerebral perfusion. Ann Thorac Surg.

[8] Ueda T, Shimizu H, Hashizume K, Koizumi K, Mori M, Shin H, Yozu R (2003). Mortality and morbidity after total arch replacement using a branched arch graft with selective antegrade cerebral perfusion. Ann Thorac Surg.

[9] Takagi H, Umemoto T (2016). A meta-analysis of total arch replacement with frozen elephant trunk in acute type A aortic dissection. Vasc Endovascular Surg.

[10] Yoshitake A, Tochii M, Tokunaga C (2020). Early and long-term results of total arch replacement with the frozen elephant trunk technique for acute type A aortic dissection. Eur J Cardiothorac Surg.

[11] Rice RD, Sandhu HK, Leake SS (2015). Is total arch replacement associated with worse outcomes during repair of acute type A aortic dissection?. Ann Thorac Surg.

[12] Shimura S, Odagiri S, Furuya H (2020). Echocardiography-guided aortic cannulation by the Seldinger technique for type A dissection with cerebral malperfusion. J Thorac Cardiovasc Surg.

[13] Fukuhara S, Norton EL, Chaudhary N (2021). Type A aortic dissection with cerebral malperfusion: new insights. Ann Thorac Surg.

[14] Nishimura M, Ohtake S, Sawa Y (2002). Arch-first technique for aortic arch aneurysm repair through median sternotomy. Ann Thorac Surg.

[15] Mills NL, Ochsner JL (1980). Massive air embolism during cardiopulmonary bypass. Causes, prevention, and management. J Thorac Cardiovasc Surg.

[16] Takano K, Yamaguchi T, Minematsu K, Sawada T, Omae T (1998). Differences in clinical features and computed tomographic findings between embolic and non-embolic acute ischemic stroke: a quantitative differential diagnosis. Intern Med.

[17] Tanaka A, Chehadi M, Smith HN (2023). Deep hypothermic circulatory arrest with retrograde cerebral perfusion: how long is safe?. Ann Thorac Surg.

[18] Settepani F, Cappai A, Basciu A, Barbone A, Tarelli G (2016). Outcome of open total arch replacement in the modern era. J Vasc Surg.

[19] Krüger T, Weigang E, Hoffmann I, Blettner M, Aebert H (2011). Cerebral protection during surgery for acute aortic dissection type A: results of the German Registry for Acute Aortic Dissection Type A (GERAADA). Circulation.

[20] Dumfarth J, Kofler M, Stastny L, Plaikner M, Krapf C, Semsroth S, Grimm M (2018). Stroke after emergent surgery for acute type A aortic dissection: predictors, outcome and neurological recovery. Eur J Cardiothorac Surg.

[21] Morimoto N, Okada K, Okita Y (2011). Lack of neurologic improvement after aortic repair for acute type A aortic dissection complicated by cerebral malperfusion: predictors and association with survival. J Thorac Cardiovasc Surg.

[22] Di Eusanio M, Patel HJ, Nienaber CA (2013). Patients with type A acute aortic dissection presenting with major brain injury: should we operate on them?. J Thorac Cardiovasc Surg.

[23] Inoue Y, Inoue M, Koga M (2022). Novel brain computed tomography perfusion for cerebral malperfusion secondary to acute type A aortic dissection. Interact Cardiovasc Thorac Surg.

[24] Gong W, Zhou L, Shang L, Zhao H, Duan W, Zheng M, Ge S (2022). Cerebral infarction and risk factors in acute type A aortic dissection with arch branch extension. Echocardiography.

[25] Okita Y, Miyata H, Motomura N, Takamoto S (2015). A study of brain protection during total arch replacement comparing antegrade cerebral perfusion versus hypothermic circulatory arrest, with or without retrograde cerebral perfusion: analysis based on the Japan Adult Cardiovascular Surgery Database. J Thorac Cardiovasc Surg.

[26] Ueda Y, Okita Y, Aomi S, Koyanagi H, Takamoto S (1999). Retrograde cerebral perfusion for aortic arch surgery: analysis of risk factors. Ann Thorac Surg.

[27] Schoonen A, van Klei WA, van Wolfswinkel L, van Loon K (2022). Definitions of low cardiac output syndrome after cardiac surgery and their effect on the incidence of intraoperative LCOS: a literature review and cohort study. Front Cardiovasc Med.

[28] Huo Y, Zhang H, Li B (2021). Risk factors for postoperative mortality in patients with acute Stanford type A aortic dissection. Int J Gen Med.

